# Reviewing the effect of teachers’ resilience and wellbeing on their foreign language teaching enjoyment

**DOI:** 10.3389/fpsyg.2023.1187468

**Published:** 2023-08-31

**Authors:** Linlin Zhang

**Affiliations:** Department of Public Basic Courses, Nanjing Vocational University of Industry Technology, Nanjing, Jiangsu, China

**Keywords:** foreign language teaching enjoyment, resilience, wellbeing, foreign language teacher, emotions, classroom environment

## Abstract

This paper reviews evidence on teachers’ resilience (TR) and wellbeing (TWB) on foreign language teaching enjoyment (FLTE). This review improves the understanding of the multi-dimensional, dynamic and context-dependent structural attributes of TR and TWB, as well as the relationship between them and the FLTE. The literature review verifies the positive effects of teachers’ positive optimism, self-efficacy, positive teacher-student relationship, teacher support and pro-social dynamic classroom environment on TR and TWB under person-context interaction, and also confirms that TR and TWB have predictive effect and significant impact on personal enjoyment, social enjoyment and student appreciation of FLTE three-factor structure. Some important findings from the review verifies the important role of teachers’ social enjoyment in the three-factor structure and the dominant role of prosocial situational characteristics in predicting FLTE. This paper finally explains its pedagogical significance and provides some suggestions for expanding the research on antecedent variables related to FLTE.

## 1. Introduction

There has recently been a significant increase in research on the emotion of foreign language (FL) learners and teachers (Dewaele et al., 2019; Dewaele, 2020a). Researchers have a strong academic interest in how to make students improve their language skills while also gain wellbeing with the help of positive teachers (Gkonou et al., 2020; Budzińska and Majchrzak, 2021), and try to find out the source of teachers’ emotional changes and adjustment strategies, as well as the impact on students’ wellbeing (Braun et al., 2020). Becker’s research found that teachers’ emotions can directly affect students’ emotions in the classroom environment (Becker et al., 2014), so the research interest for FL teachers’ wellbeing (Mercer and Gregersen, 2020) and resilience (Hiver, 2018; Kostoulas and Lammerer, 2020) has also been growing rapidly recently. Wellbeing and resilience are central to the teachers’ FLTE because they can create a positive classroom environment for learners to enjoy themselves and make progress (Moskowitz and Dewaele, 2019), which are essential for high-quality education and learning outcomes (Li et al., 2021). However, due to the multidimensional, dynamic and context-dependent nature of resilience and wellbeing, as well as the complexity of person-context interaction, the relationship between resilience, wellbeing and FLTE, especially the influence of classroom environment on EFL teachers’ personal enjoyment, social enjoyment and students’ appreciation, has not been fully investigated. Therefore, it is very important for both teachers’ and students’ FL enjoyment and mental health to have in-depth knowledge and research on the antecedents of promoting teachers’ FLTE.

The exploration of this review may encourage teachers to realize that the construction of positive emotions, the creation of positive classroom environment and pro-social classroom behaviors in teaching practice will help reduce job burnout and strengthen TR, thus bringing beneficial effects on the FLTE. Meanwhile, students can also experience the wellbeing and FL enjoyment brought by teachers with high FLTE level. Some important findings in the review provide valuable insights into future FLTE research. The research suggestion on expanding the antecedent situation variables of FLTE can increase the possibility for researchers in this field to further explore the study of FLTE, which is of great significance for further enriching the structure of FLTE.

## 2. Methods

### 2.1. Literature search

The purpose of this study was to explore the influence of TR and TWB on FLTE. Web of Science - SSCI, Elsevier ScienceDirect (SD) and Springer databases were searched exhaustively in February 2023. A computerized literature search was conducted to find articles published in English between 1990 and January 2023. These articles use “resilience,” “wellbeing,” and “foreign language teaching enjoyment “as keywords or terms in the title or abstract or topic, with the following expressions: “teacher,” “emotion,” “personality,” “emotional intelligence,” “adaptability,” “stress,” “self-efficacy,” and “job satisfaction.” All studies consider the relationship between keywords and these terms of expression. In addition, the bibliography was searched manually so that the database can be supplemented. All articles referenced are peer-reviewed journals, conference proceedings, and doctoral dissertation articles.

### 2.2. Inclusion and exclusion criteria

Articles that meet the following inclusion criteria was included in my review. The first criterion for inclusion is that the article should be an empirical study, which excluded theoretical studies and reviews. The second criterion is that the paper includes TR or TWB as relevant variables. Considering the multi-dimensionality of the two variables and the complexity of their effects on enjoyment, factors closely associated with these two variables, such as “emotion,” “personality,” “emotional intelligence,” “adaptability,” “stress,” “self-efficacy,” and “job satisfaction,” are also taken into account. That is, if two variables or one of them co-occur with any of these factors, they have been included in the review. The third criterion is that the review is a survey of teaching professionals, so I excluded studies of groups such as hotel management, corporate employee management, and healthcare workers. In addition, teaching staff in disciplines other than foreign languages were not included in my study. The fourth criterion is that my assessment framework for FLTE is based on its three-factor structure, that is, teachers’ personal enjoyment, social enjoyment and students’ appreciation, and the classroom environment is the main situational place for implementing the assessment. Therefore, I excluded articles that studied other occupational environment factors (such as institutions, schools, organizations, etc.).

### 2.3. Study selection

After a preliminary search, I identified 218 potentially eligible studies: 4 in Web of Science-SSCI, 104 in Elsevier ScienceDirect, and 110 in Springer. After eliminating duplicates, 156 relevant reports were obtained. During this phase, researchers screened titles and abstracts based on inclusion and exclusion criteria. The reasons for the exclusion of this step are mainly due to (1) not including TR or Wellbeing as variables, (2) not based on the three-factor structure of FLTE assessment, (3) non an empirical research, and (4) not using teaching professionals as samples. Sixty-two articles included in the criteria were retained after the screening. The researchers then manually searched the list of references for these articles to find other research articles with high correlations between the two principal variables and other factor variables, and came up with 26 more. All these included articles serve as the main references for my analysis and comment on the impact of TR and TWB on FLTE.

## 3. Literature review

### 3.1. Teacher resilience

There have been many studies on Teacher resilience (TR) over the past 20 years (e.g., Brunetti, 2006; Day and Gu, 2009; Beltman et al., 2011; Gu and Li, 2013; Bowles and Arnup, 2016; Gu, 2018; Fan et al., 2021). Its basic ideas are summarized in three factors: quality, outcome and process.

The definition of quality theory defines TR as an individual’s own quality or ability (Brunetti, 2006). Teachers with resilience have the ability to bounce back continuously in the face of adversity and recover their strength or spirit quickly and effectively (Gu and Day, 2007), maintain their commitment to teaching or have a professional wellbeing (Brunetti, 2006). This ability to “bounce back” quickly is an important quality for teachers to keep growing and has been widely recognized in the conceptualization of TR (Connor and Davidson, 2003; Oswald et al., 2003; Pretsch et al., 2012; Leroux, 2018).

The outcome theory defines TR is the outcome of teachers’ positive adaptation in adversity (Masten and Obradovic, 1994). According to this definition, teachers can effectively use supportive resources in the school environment to achieve goals and thus achieve good adaptation when facing adverse environment. Therefore, resilient teachers tend to be more confident and optimistic, and have a sense of achievement and wellbeing (Petterson et al., 2004), but rarely mentions how individuals adjust themselves to adapt to the environment. Masten and Obradovic (2006) believe that psychological resilience is a situation in which individuals can continue to develop well after negativity and setbacks.

Researchers holding the view of dynamic process theory (e.g., Gu and Day, 2007; Gu, 2018; Li et al., 2019) believe that TR is a relative dynamic and interactive process of positive adaptation and personal development under challenging conditions. This interactive process is dynamically influenced by teachers’ personality, professional mission, values, school leader, colleague relationship and the significant others (Sammons et al., 2007; Day and Gu, 2009). This kind of process definition is obtained on the basis of the above two factors, which is more recognized in academia.

In the study of teacher resilience structure, most studies have found that TR is a multi-dimensional structure, and various factors cross each other (Luthar et al., 2000; Gu and Day, 2007, 2011; Mansfield et al., 2012, 2016; Gu and Li, 2013). For example, Mansfield et al. (2012) proposed a four-dimensional TR framework, namely, career-related, emotional, social and motivational. Some researchers proposed that career-related factors include teachers’ professional decision-making, self-insight, professional freedom, initiative (Sumsion, 2004), attention to students’ learning (Petterson et al., 2004), and use of coping strategies (Sharplin et al., 2011). The idea that emotion in this framework is an important attribute of TR has also received academic support from other researchers, such as strong sense of competence, efficacy, achievement and sense of humor (Bobek, 2002), emotional intelligence (Bardach et al., 2021), etc., are all very important emotional factors in the construction of TR. Some researchers have pointed out that the mutually supportive interpersonal relationship, professional relationship and peer relationship in the social dimension are also important factors in the process of developing TR (Sammons et al., 2007). Beltman et al. (2011) constructed the structural framework of TR from the two dimensions of protective factors and risk factors. Both protective factors and risk factors come from both individual and environment aspects, and work together in a complex and dynamic way to shape TR (Beltman et al., 2011). In terms of resilience measurement, Mansfield and Wosnitza (2015) developed a multidimensional TR table and applied it in different educational situations (Peixoto et al., 2018).

As can be seen from the above literature studies, the definition of TR has not yet reached a consensus. This is because the concept of resilience has distinct cultural connotations. People in different countries, races and regions may interpret it differently due to the influence of cultural customs and social development level (Ungar et al., 2008). The review also reveals that TR is a dynamic structure established by person-context interaction, and has the characteristics of complex and multi-dimensional structure. Studies in the literature have linked TR with factors such as personal motivation, emotion, self-efficacy, professional adaptability and interpersonal relationship, and found that intrinsic motivation and self-efficacy are important personal protective factors, which can enable teachers to maintain adequate resilience and staying power under challenging circumstances. While protective school environment factors contribute to TR and their teaching commitment, and can buffer or weaken teachers’ burnout experience and improve their wellbeing. However, the research on intervention and measurement of TR is still a little insufficient, such as designing effective and practical TR intervention measures to promote good social adaptation and healthy development of individuals, and how to develop a TR scale suitable for local characteristics according to cultural and individual differences between countries.

### 3.2. Teacher wellbeing

Wellbeing is a complex structure, and defining it can be challenging (Dodge et al., 2012). In the current literature, it is generally accepted that wellbeing is conceptualized from the three main characteristics of the structure: multi-dimensionality, dynamic nature and context-dependent. For example, Dzuka and Dalbert (2007) divided teacher wellbeing (TWB) into three dimensions: overall life satisfaction, positive emotion and negative emotion, which belong to the category of subjective wellbeing. As a sign, the study of TWB has entered the stage of multi-disciplinary concept definition, and presents the characteristics of multi-dimensional structure. de Albuquerque et al. (2011) recruited teacher groups as research participants to verify the three-dimensional structure of subjective wellbeing, and explicitly included the concept of “subjective wellbeing” into the research field of TWB. Oxford (2016) proposed the empathy model assuming that a person’s wellbeing is achieved through the interplay of nine factors: namely emotion and empathy, meaning and motivation, perseverance, agency and autonomy, time, hardiness and habits of mind, intelligence, character strengths, and self-related factors. Seligman (2011) put forward a multi-componential model of wellbeing for realizing a happy life by combining hedonic and eudemonic perspectives. The five-element model (PERMA) includes positive emotions, engagement, relationships, meaning, and accomplishment. Therefore, it has been widely recognized that wellbeing is a complex multidimensional structure composed of multiple components (Dodge et al., 2012; Hascher and Waber, 2021). According to Butler and Kern (2016), there is no optimal wellbeing model, but different conceptualizations help to discuss the abstract construction of wellbeing and provide measurable, developed and sustained concrete domains.

Wellbeing is also considered to have some characteristics of complex dynamic systems, such as dynamics, interconnectedness and situatedness (e.g., Oxford, 2018; Lomas et al., 2020). Day and Gu (2010) demonstrated that TWB is generated by the complex interaction of personal, professional and situational factors at specific points participants’ life and career. Positive and negative emotions can also be experienced simultaneously by individuals in their daily lives (Trampe et al., 2015). Boudreau et al. (2018) examined the fluctuating relationship between positive emotions (enjoyment) and negative emotions (anxiety) within individuals by using oral interviews and story-telling task in a controlled setting, and found that the relationship was highly dynamic, its changes can occur rapidly in days or hours or even minutes, which means that wellbeing is dynamic on multiple time scales along with the interaction between the individual and the environment. Despite the transient and fluctuating nature of positive emotion, it can have a long-term impact on enhancing wellbeing (Garland et al., 2010). Insight into the dynamic mechanism of TWB in FL teaching classrooms is also of great significance for creating a dynamic prosocial classroom atmosphere and intervention applications. However, to date, only a few studies have focused on two or three discrete antecedents of teacher emotion or TWB using instantaneous methods in the classroom (e.g., Becker et al., 2015; Frenzel et al., 2015; Goetz et al., 2015). In terms of the measurement methods of TWB, the vast majority of studies take TWB as a trait (Hascher and Waber, 2021), while only a few studies used daily measurements for dynamic assessment of TWB (e.g., Simbula, 2010; Tadić Vujčić et al., 2017; Aldrup et al., 2018; Lavy and Eshet, 2018).

As can be seen from the above, TWB is a complex structure with multidimensional, dynamic and context-dependent attributes. At present, there is no consensus on the definition of TWB, and the measurement methods and dimensions of TWB are not clear and comprehensive, which may hinder the development of concise TWB theory and good practice of using evidence-based knowledge. In order to promote the research progress of TWB, it is necessary to strengthen the establishment of TWB concept system, so that researchers can study TWB systematically under the same language family. In addition, the academic circle should also promote the compilation of TWB scale and questionnaire with scientific authority, so as to improve the reliability and validity of TWB quantitative research.

### 3.3. Foreign language enjoyment and foreign language teaching enjoyment

Enjoyment is a kind of positive emotion that differs from the basic pleasure experience, which includes dimensions of intellectual focus, heightened attention, and optimal challenge (Boudreau et al., 2018), that is, enjoyment can enhance learners’ attention and their awareness of language features, and helps them consciously pay attention to, process and better acquire a target language (Dewaele and MacIntyre, 2016; Dewaele and Alfawzan, 2018). In terms of foreign language learning, Dewaele and MacIntyre (2016) put forward the concept of foreign language enjoyment (FLE) and argued that FLE is a complex emotion related to the interaction between challenge and perceptual ability, and further points out two dimensions of FLE, namely social and private dimension. The social dimension focuses on friendly classroom environments, interactions with supportive peers, emotional support and encouragement from teachers, as well as stimulating and challenging classroom activities, thus resulting in positive emotions and satisfaction (Dewaele and MacIntyre, 2016; Dewaele and Li, 2022). The private dimension involves the perception that links enjoyment, achievement, creativity and challenge, as well as an inner pride and success in the face of challenges and obstacles (Dewaele and MacIntyre, 2016; Pavelescu and Petrić, 2018; Dewaele and Li, 2022). Some studies have emphasized the role of enjoyment in foreign language learning. For example, Dewaele and MacIntyre (2014) found that learners’ enjoyment level was higher than their anxiety level, and believed that learners’ enjoyment level was related to their education level and foreign language level. They also suggest that the power of FLE may enhance intrinsic and extrinsic motivation to learn a foreign language and increase students’ awareness of language input. Dewaele and Alfawzan (2018) also found that the degree of enjoyment is significantly correlated with the overall score of learners’ foreign language skills. Another of their studies (Dewaele and Li, 2022) also found that learners’ foreign language classroom anxiety and FLE were associated with more self-perceived general English proficiency and less actual English achievement.

The concept of foreign language teaching enjoyment (FLTE) was proposed by Proietti Ergün and Dewaele (2021), which is a positive emotion mirroring learners’ FLE. FLTE refers to the teachers’ happiness, pleasure and wellbeing in teaching foreign languages and the teachers’ ability to provide a pleasant classroom atmosphere (e.g., Mierzwa, 2019; Proietti Ergün and Dewaele, 2021). According to Proietti Ergün and Dewaele (2021), FLTE consists of three subdomains including personal enjoyment, social enjoyment and learners’ appreciation in the classroom. Ensuring the FL teachers’ wellbeing and enhancing their resilience help them to radiate happiness and boost their enjoyment of teaching (MacIntyre, 2021; Proietti Ergün and Dewaele, 2021). Echoing these ideas, in a study of 129 adult students worldwide, Moskowitz and Dewaele (2019) found that happy teachers were more likely to create a happy classroom for their students, thus revealing a strong link between classroom environment (CE) and positive emotions.

Foreign language teaching enjoyment is a positive emotion that plays a key role in the career of FL teachers. According to the study of Proietti Ergün and Dewaele (2021), teachers with high levels of FLTE have higher job satisfaction and wellbeing. In other words, teachers of this kind who are mentally healthy and happy are less likely to experience burnout and emotional exhaustion. Therefore, it is necessary to conduct in-depth research on the related factors affecting FLTE. The two main factors that affect FLTE are emotional regulation and mental health. Language teachers who are able to control, manage or regulate their emotions enjoy higher levels of work efficiency, job engagement and wellbeing (Bielak and Mystkowska-Wiertelak, 2020; Greenier et al., 2021); psychologically happy and resilient language teachers can sustain their positive feelings despite daily stressors (Wang et al., 2022). Especially in the face of adversity, the successful use of emotion regulation strategies can obtain a higher level of wellbeing, dedication and self-efficacy (Azari Noughabi and Amirian, 2020; Noughabi et al., 2020), so that they can show better FLTE.

The above research on FLTE literature shows that FLTE is a kind of positive emotion that plays a key role in teachers’ career. Its power can not only maintain teachers’ mental health and enable them to enjoy teaching, but also bring students wellbeing and enjoyment in learning, and improve their academic performance by providing a positive classroom atmosphere. Therefore, FLTE is closely related to teachers’ personal enjoyment, social enjoyment and learners’ appreciation in the classroom, and is a positive result of person-context interaction.

## 4. The influence of TR on FLTE

Mierzwa (2019) was the first researcher to apply the FLE scale (Dewaele and MacIntyre, 2014) for teachers. The study subjects were 89 Polish FL teachers with different levels of education and rich teaching experience. The study found that Polish FL teachers had a high level of enjoyment in teaching, and all the survey variables, including gender, place of residence, years of experience in the major, type of school and type of language taught, had no effect on teachers’ FLTE. It was demonstrated that the teacher’s positive attitude toward students, the positive classroom atmosphere created, and the useful and interesting course content provided to students resulted in the students’ FLE.

Proietti Ergün and Dewaele (2021) examined the construction of FLTE with a sample of 174 Italian FL teachers. The test scale adopted was the adjusted short table of 9 items and 3 factors developed by Botes et al. (2021). The three factors include teachers’ personal enjoyment, social enjoyment and students’ appreciation in the classroom. Simplified FL enjoyment scale (S-FLES) developed by Botes et al. (2021) is developed on the basis of the FL enjoyment scale (FLES) proposed by Dewaele and MacIntyre (2014). The results show that there is a positive correlation between FL teachers’ resilience, wellbeing and FL learning ability. Teachers’ resilience and wellbeing were both significant predictors of FLTE, but resilience was stronger than wellbeing in predicting FLTE. This indicates that FL teachers with happy psychology and resilience are able to regulate and control their emotions and are more likely to accomplish their professional goals which leads them to more energy, love and enjoyment (Greenier et al., 2021), and are fully capable of providing students with a positive classroom atmosphere and make contributions to students’ FL learning and psychological growth. In the study of demographic data variables, the results of other FLTE are very similar to the results of Mierzwa (2019) study on Polish teachers, except that the type of school for teachers significantly affects their resilience and FLTE.

Echoing the findings of Proietti Ergün and Dewaele (2021) and Derakhshan et al. (2022) measured the effects of resilience, wellbeing, and second language grit on FLTE of 450 Iranian FL teachers using the S-FLES scale adjusted by Proietti Ergün and Dewaele (2021). The results showed that grit, resilience, and wellbeing of FL teachers were significant predictors of FLTE, while second language grit was found to be the strongest predictor of FLTE. Meanwhile, they also found that there were significant correlations between grit and resilience, and between resilience and wellbeing, and teachers’ resilience had a significant impact on their FLTE. The results also confirmed a significant association between teachers’ grit and their mental health, indicating that teachers with tenacity are full of passion for achieving professional goals, and their strong desire for achievement and success makes them less likely to experience burnout (Sudina et al., 2020). As a result, gritty teachers are more likely to achieve wellbeing and joy. A strong correlation between FL teachers’ resilience and FLTE (Proietti Ergün and Dewaele, 2021; Derakhshan et al., 2022) showed that as a FL teacher, not being hurt seems to be more important than enjoying, and resilience can play a protective role in mental health and wellbeing, and promote FLTE (Proietti Ergün and Dewaele, 2021). Moreover, teachers’ resilience and coping strategies can help them achieve wellbeing and satisfaction (MacIntyre et al., 2020).

These important findings by Proietti Ergün and Dewaele (2021) as well as Derakhshan et al. (2022) confirm previous research that resilient teachers have the ability to bounce back quickly and regain their strengths in the face of professional adversity, and maintain the commitment to teaching or have professional wellbeing (Brunetti, 2006; Gu and Day, 2007). Such a teacher is more determined to overcome stressors and maintain interest even when faced with setbacks (Hiver, 2018). Moreover, the resilience of persevering teachers helps them maintain their professional commitment and enthusiasm in the face of professional adversity (Liu and Chu, 2022). Similar to the partial findings of Derakhshan et al. (2022), a study completed by Liu and Chu (2022) also identified the three-factor structure of Chinese EFL teachers’ resilience, including tenacity, optimism and coping style. It is found that the overall resilience of EFL teachers is at a medium high level. Among the three dimensions of resilience, tenacity is at a higher level, followed by optimism and coping style. The study reflected teachers’ positive emotions and positive expectations related to their wellbeing in the presence of difficulties, indicating a significant correlation between resilience and wellbeing of FL teachers. This is because optimism and self-efficacy are prominent characteristics of resilient teachers (Gu and Li, 2013; Li et al., 2019), resilient teachers are capable of adopting regulating strategies to maintain positive emotions and cope with stressors (Mansfield et al., 2012), and are more likely to experience wellbeing and flourish throughout their careers (Hiver, 2018; MacIntyre et al., 2019).

For FL teaching, the teacher’s FLTE is mainly reflected in the personal enjoyment, social enjoyment and learners’ appreciation in the classroom, which reflects the teacher’s ability to provide a pleasant classroom atmosphere (Proietti Ergün and Dewaele, 2021). In the existing literature, the influence of TR corresponding to the three dimensions of teaching enjoyment is mostly reflected in the teacher’s emotional factors, interpersonal relationship and classroom environment.

The emotional factor is the inner driving force of the formation of TR and plays an important role in teachers’ personal enjoyment. The sense of joy, pride and achievement enjoyed by teacher in the classroom symbolizes the experience of positive emotions in the individual. Fredrickson (2004) mentioned in her broaden-and-build theory of positive emotions that happiness, interest, satisfaction and love, which are important reserve resources of individuals, can all be components of positive emotions, and can effectively promote the development of resilience. In other words, positive emotions can increase TR. If positive emotion can be effectively explored, health, subjective wellbeing and resilience can be optimized (Rahimi and Bigdeli, 2014). Therefore, teachers should consciously accumulate and reserve personal resources of such positive emotions to improve their level of personal enjoyment in the classroom, so as to accumulate strength for resilient development. Strong sense of competence, efficacy, accomplishment and sense of humor (Bobek, 2002), emotional intelligence (Bardach et al., 2021), etc., are very important positive emotional factors to construct TR.

Interpersonal relationship is a necessary resource condition for TR development and plays an important role in teachers’ social enjoyment. For example, teachers establish important relationships with students, colleagues, leaders, family and friends as well as professionals (Le Cornu, 2013). Harmonious teacher–student relationship is a key factor affecting TR and professional commitment (Gu, 2018). It will promote teacher self-efficacy (Zee et al., 2017), emotional wellbeing (Milatz et al., 2015), and job satisfaction (Admiraal et al., 2019), and increases teacher motivation, effort, participation, happiness and confidence, which in turn may lead to greater use of complex, high-impact teaching practices by teachers (van der Lans et al., 2020). In addition, mutual support and common teaching goals among teachers are conducive to maintaining their resilience (Ellison and Woods, 2018), and the support of schools and school leaders also plays an indispensable role in sustaining teachers’ sense of resilience (Day and Hong, 2016; Gu, 2018). Through the mutual trust between teachers and students, good relationship with colleagues, the support of the school and leaders, as well as the encouragement and comfort of family members, teachers can improve their psychological resilience in adversity, better cope with setbacks, and maintain their professional enthusiasm and teaching passion in difficulties. Therefore, positive interpersonal relationship can enhance teachers’ inner sense of pleasure, efficacy and wellbeing, so as to improve teachers’ level of social enjoyment.

A pleasant classroom atmosphere not only enables FL teachers and learners to enjoy teaching and learning at the same time, but also serves as an important situational factor for teachers to win the appreciation of students. Students’ appreciation comes more from their perception of teachers’ enthusiasm, the happy classroom atmosphere provided by teachers and their enjoyment of learning. Only when FL teachers are mentally healthy, resilient and indomitable can they handle the daily teaching challenges and enjoy teaching well, and have the ability to create a positive emotional atmosphere for students in the class and bolster their happiness and linguistic development (Proietti Ergün and Dewaele, 2021; Derakhshan et al., 2022). Studies have shown that self-efficacy is an important motivational dimension of TR structure (Mansfield et al., 2012) and plays an important role in classroom management, teaching behavior and learning enjoyment (Hettinger et al., 2021). Teachers’ self-efficacy in classroom management is positively correlated with the supportive atmosphere in the classroom and predicts a higher level of teacher-student intimacy (Zee et al., 2017). Positive classroom environments are associated with positive affective outcomes, including interest, enjoyment, emotional competence, motivation, comfort, and positive attitudes toward subject and participation (Dorman and Fraser, 2008). Therefore, resilient teachers with positive emotions have the ability to provide a happy classroom atmosphere for students to enjoy learning, and students’ appreciation in turn promotes teachers’ personal and social enjoyment.

The above literature review confirmed that TR had a significant effect on their FLTE and was a stronger predictor of FLTE than TWB. It shows that TR can protect TWB and their mental health, but also promote teachers’ FLTE. Resilient teachers maintain their professional commitment and passion for teaching or have professional wellbeing even in the face of adversity. Meanwhile, it also proves that positive emotion can promote TR, which is an important individual reserve resource conducive to the development of TR and plays an important role in the personal enjoyment of teachers. Positive interpersonal relationship is the necessary resource condition for TR development and plays an important role in teachers’ social enjoyment. Resilient teachers also have the ability to provide students with a happy classroom atmosphere, thus enabling teachers to gain personal and social enjoyment as well as students’ appreciation.

## 5. The influence of TWB on FLTE

It has been demonstrated that TWB is a significant predictor of teaching enjoyment (Proietti Ergün and Dewaele, 2021; Derakhshan et al., 2022). TWB is the result of the interaction between individual emotion and different situational factors, which is manifested as a positive mental state and a series of positive behaviors related to it. In the context of FL teaching, the influence of TWB on the three dimensions of personal, social and student appreciation (Proietti Ergün and Dewaele, 2021) in the structure of FLTE is mainly reflected in the research on the prediction and influencing factors of TWB, including individual internal factors of teachers and classroom environment (CE) factors.

Teachers’ individual internal factors are mainly studied from the theoretical perspective of psychology to investigate the influence of teachers’ internal characteristics on their wellbeing. The internal factors that have been paid more attention in the literature mainly include emotion, intrinsic motivation, mindfulness, emotional intelligence, personality traits, self-management and evaluation, creativity, etc., which affect TWB and their personal enjoyment in teaching.

The broaden-and-build theory of positive emotions proposed by Fredrickson (2001, 2004), Fredrickson and Joiner (2018) focuses on capturing the influence of positive emotions. Positive emotions are a sign of optimal wellbeing, it can promote wellbeing and prosperity not only when experiencing positive emotions, but also after experiencing them. Positive emotions broaden people’s minds and horizons, help eliminate the lasting effects of negative emotional arousal, and stimulate effective responses to stressful experiences. This promotes resilience, builds personal and intellectual resources, and promotes personal wellbeing (MacIntyre and Gregersen, 2012). Frenzel (2014), in his review of teachers’ emotions, identified enjoyment as one of the main positive emotions experienced by teachers at work, which represents wellbeing and joy. Seligman (2011) proposed the influential five elements model (PERMA) for realizing a happy life, among which positive emotion is an important element of happiness. Therefore, there is every reason to believe that positive emotions can promote FL teachers’ personal enjoyment and help them build personal resources for resilience and wellbeing (Nalipay et al., 2019; Johnson et al., 2021), such healthy personal emotional resources can be extracted when coping skills are needed to successfully overcome a specific situation. Daily positive emotions increased the likelihood of individuals experiencing wellbeing in the future, as far as 5 weeks (Fredrickson and Joiner, 2018).

Self-efficacy is a person’s belief of confidence that they can successfully perform a given task. According to self-determination theory (Deci and Ryan, 2000), it is one of three factors impacting intrinsic motivation. It captures the core aspect of human agency and is reflected in efforts and persistence to achieve expected goals. In the framework of teacher motivation proposed by Tschannen-Moran and Hoy (2001), the role of teacher self-efficacy in the development of teachers and students is emphasized. For teachers, self-efficacy increases the persistence of working with challenging students and has been shown to influence teachers’ teaching practices, enthusiasm, commitment, and teaching behavior (e.g., Skaalvik and Skaalvik, 2007; Burić and Kim, 2020). Teachers’ self-efficacy in classroom management is positively correlated with supportive classroom atmosphere and can predict a higher level of teacher-student relationship intimacy (Zee et al., 2017). It can be considered that self-efficacy can enhance classroom management and positive teacher-student interaction. Teachers will experience personal enjoyment in the process of intimate interaction with students, and students’ perceived teacher support is also related to students’ subsequent enjoyment of learning (Lazarides and Buchholz, 2019).

Mindfulness, emotional intelligence and personality traits also had positive effects on teachers’ mental health and wellbeing. A systematic review by Hwang et al. (2017) shows that mindfulness intervention can effectively reduce teachers’ stress, anxiety, burnout and depression, thus improving TWB. Dewaele et al. (2018) found that there was a positive correlation between teachers’ emotional intelligence, teaching experience, proficiency and teaching practice. They believe that developing English teachers’ emotional intelligence can improve their love of English, teaching skills and career wellbeing. MacIntyre et al. (2019) investigated the wellbeing of language teachers based on the PERMA model of Seligman (2011) and the Big Five personality model of Goldberg (1990). They show that TWB can be positively predicted by teachers’ perceive positive emotion, engagement, positive relationship, meaning and achievement. They found positive correlations between language teachers’ wellbeing, agreeableness, conscientiousness, emotional stability and intelligence. Accordingly, teachers’ positive traits such as theology, restraint and interpersonal virtues, as well as character strengths such as optimism and social responsibility, are positively correlated with TWB (Kim and Lim, 2016).

Mattern and Bauer (2014) studied the relationship between self-management and TWB. The results showed that teachers could reasonably plan time through self-management, reduce emotional exhaustion, and thus improve job satisfaction and wellbeing. While teachers’ self-positive evaluation of their teaching situation (such as feeling satisfied with working conditions, feeling competent, or feeling committed to school) was positively correlated with at least some aspects of TWB, negative evaluation (such as feeling stressed or burdened, overworked) was negatively correlated with some aspects of TWB. Both sense of competence and sense of teaching efficacy are significantly positively correlated with TWB (e.g., Vazi et al., 2013; Capone and Petrillo, 2018). Other studies have explored the relationship between creativity and TWB. For example, Tan and Majid (2011) found that teachers with higher creativity have higher TWB, while Jennings (2014) showed that TWB also affects teachers’ creativity. It can be seen that the two are mutually promoting relations. In addition, some studies hold that TR is also closely related to TWB. For example, Pretsch et al. (2013) believe that TR can predict TWB level.

In terms of CE factors, literatures mainly focus on the factors that predict and affect TWB, including classroom, teacher-student relationship and interaction, student participation and investment, and teacher support. All these factors affect teachers’ FLTE, and students’ appreciation of their teachers is mainly reflected in the perception and attitude of teachers’ positive teaching behavior in the interaction process of personal emotion and classroom teaching situation.

Classroom is a direct scene for teachers and students to experience emotions together, and also an important place for them to gain wellbeing and FLE. The accumulation of common emotional experience between teachers and students establishes the CE. A positive CE is a positive emotional tone associated with friendliness, caring, encouragement, cooperation, cohesion, mutual support and respect, appropriate competition, and positive interaction (Harvey et al., 2012). Jennings and Greenberg (2009) pointed out that teachers with high social and emotional competence and wellbeing are more likely to establish and maintain teacher-student rapport and create a positive CE that promotes learning and student development. Khajavy et al. (2017) found that a positive CE was related to fostering student enjoyment and willingness to communicate while reducing anxiety. Especially when students have positive cognition of CE, including emotional support, teaching support and good classroom organization, students have higher enjoyment, lower anxiety and higher willingness to communicate. Therefore, in a positive social psychological environment, positive feelings such as enjoyment, comfort and interest can be easily aroused, which will benefit teachers’ social enjoyment and students’ learning enjoyment, as well as students’ appreciation of a pleasant classroom atmosphere.

The development of healthy teacher–student relationships has always been the focus of educational research (Hughes, 2011), and the relationship between emotions and classroom in the social psychological dimension (i.e., teacher–student relationships and peer relationships) has always been considered as two core characteristics of the CE (Harvey et al., 2012). This is due to the benefits of prosocial dynamics in the CE. For students, teachers characterized by warmth, trust, respect and closeness will not only be favored and appreciated by them, but also have a positive impact on their academic performance (Li, 2017) and psychological adjustment (Cornelius-White, 2007). For teachers, such prosocial classroom behavior can also protect their own mental health and promote their perception of professional competence by reducing burnout and negative emotions (Klassen et al., 2012). Prosocial behavior also enables them to benefit from positive teacher-student interaction. When students interact with teachers, they develop a stronger academic self-concept (Kim and Sax, 2014) and report higher confidence (Micari and Pazos, 2012). A supportive CE increases students’ sense of belonging, engagement and motivation (Zumbrunn et al., 2014). Studies have shown that interaction with friendly peers, supportive and encouraging teachers, and a positive, interesting and challenging CE are positive mediators for FLE (Dewaele and MacIntyre, 2014; Pavelescu and Petrić, 2018). Therefore, positive teacher-student relationship plays an important role in TWB, which can support TWB (Aldrup et al., 2018), and is also an important way for teachers to win students’ appreciation and social enjoyment.

Students’ classroom participation is a dynamic multi-dimensional structure, which is related to students’ classroom behavior. For students, the construct of participation involves situated notions of cognition, affect and behaviors including social interactions, in which action is a necessary component (Hiver et al., 2021). Thus, the concept of participation includes positive feelings, motivation and, above all action. When students are immersed in teaching, engagement is also an indicator of student motivation (Reeve et al., 2004), which is related to attention, flexible thinking and enjoyment, and focus on the task of interest. The three main components of engagement are behavioral, emotional and cognitive engagement (Fredricks et al., 2004). Mercer (2019) confirms that participation is the key to successful language learning. This is a strong affirmation of the vital importance of supporting learner investment in learning. The study of Botes et al. (2021) shows that there is a significant positive correlation between enjoyment, participation, motivation intensity and learners’ willingness to communicate. In the model proposed by Frenzel (2014), teachers’ perception of students’ behavior shapes their emotions in the classroom. When teachers perceive higher levels of student engagement in line with their goals, they may experience positive emotions such as enjoyment. Conversely, teachers may experience negative emotions such as anger if they perceive that student engagement is low and inconsistent with their goals. Therefore, students’ classroom participation and emotional input will affect teachers’ personal and social enjoyment.

Teacher support is one of the most important sources of social support for young students and plays a protective role in student development (e.g., Ma et al., 2017). If students perceive that teachers support and care for them, they are more likely to believe in their ability to learn and to gain positive academic emotions. Studies have found that teachers’ support for students is positively correlated with students’ academic emotions, such as enjoyment (Lei et al., 2018), while academic emotions are positively correlated with academic achievement (Ma et al., 2017). Therefore, teacher support has additional and independent contributions to students’ emotional, behavioral, and cognitive development, suggesting that teacher support may compensate for the lack of family or peer support (Quin et al., 2018). In the model of understanding teachers’ motivation proposed by Butler (2012) includes, in addition to teaching specific goals, social goals that reflect teachers’ motivation to establish a caring relationship with students. Empirical findings on social goals further illustrate the critical importance of assessing teachers’ motivations in developing supportive and caring relationships with students. Wang et al. (2016) found that teachers who reported stronger social goals also reported higher levels of teaching-related enjoyment, echoing the findings of Butler (2012) that teachers’ social goals were consistently found to more strongly predict better teaching practices and TWB. Therefore, teacher support can play a good predictive role in both learners’ and teachers’ enjoyment.

The above literature review suggests that TWB is a significant predictor of FLTE. TWB is the result of interaction between individual emotion and different situational factors. Among them, positive emotions are the hallmark of the best TWB, which can promote the personal enjoyment of teachers and help them build up TWB’s personal resources. The variable of self-efficacy can predict a higher level of teacher-student intimacy, so that teachers can easily experience personal and social enjoyment from the intimate interaction between teachers and students. Other internal factors, such as mindfulness intervention, emotional intelligence, positive personality strengths and creativity, also have a positive impact on teachers’ mental health, wellbeing and FLTE.

Combined with the influence of TR on FLTE, it can be seen that the social enjoyment subdomain dominates the overall FLTE and is more closely related to the prosocial classroom context than personal enjoyment and student appreciation. Many prosocial behaviors, such as teacher-student relationship, peer relationship, students’ participation and investment, and teacher support, are closely related to active classroom context. Personal enjoyment mainly symbolizes the experience of individual positive emotions; Students’ appreciation mainly comes from their perception of teacher’s enthusiasm and subsequent participation and investment. This means that situational factors are more important than individual characteristics in predicting FLTE, and prosocial situational factors dominate FLTE prediction.

## 6. Implications and suggestions

TR and TWB are very important to their mental health and teaching growth. This review examines the influence of TR and TWB on FLTE, based on the three subdomains of FLTE proposed by Proietti Ergün and Dewaele (2021), namely, individual enjoyment, social enjoyment and learner appreciation in the classroom. Considering the general characteristics of language teaching, the concept of FLTE in this review can also be applied to other FL teaching. As the FLTE is a new concept put forward in recent years, there are few empirical studies on its structure. From the dynamic, multi-dimensional and context-dependent attributes of TR and TWB, this review confirms the three-factor structure of FLTE under person-context interaction, and obtains some important findings from it. The key points are summarized in Table 1. The literature review shows that social enjoyment in the three-factor structure plays a more important role in the overall FLTE, which is closely related to the classroom environment. There are more person-context prosocial interaction behaviors, such as teacher-student relationship, peer relationship, student participation and teacher support, and its situational characteristics should play a dominant role in predicting FLTE. In this sense, the research suggestion of expanding the situational antecedent variables in FLTE proposed in this review can increase the possibility for researchers in this field to further explore FLTE, which is of great significance for further enriching the structure of FLTE.

**Table 1:** Summarize the main points of the review.

Main Findings	Examples (most recent publication)
TR and TWB are significant predictor of FLTE.	Proietti Ergün and Dewaele (2021), Azari Noughabi et al. (2022), and Derakhshan et al. (2022)
FL grit is a stronger predictor of FLTE than TR and TWB, There are significant correlations between grit and TR, and TR and TWB.	Derakhshan et al. (2022)
Resilient teachers are capable of adopting regulating strategies to maintain positive emotions and cope with stressors.	Mansfield et al. (2012), Hiver (2018), and MacIntyre et al. (2019)
Positive emotions can optimize TR and TWB and improve the personal enjoyment level of teachers.	Bobek (2002), Rahimi and Bigdeli, 2014, Bardach et al. (2021), and Johnson et al. (2021)
Interpersonal relationship is a necessary resource condition for TR development and plays an important role in teachers’ social enjoyment.	Milatz et al. (2015), Zee et al. (2017), Ellison and Woods (2018), Gu (2018), and Admiraal et al. (2019)
A pleasant classroom atmosphere can make both teachers and students enjoy and is also an important situational factor to win the appreciation of students.	Dorman and Fraser (2008), Zee et al. (2017), Proietti Ergün and Dewaele (2021), and Derakhshan et al. (2022)
Mindfulness, emotional intelligence and personality traits also had positive effects on teachers’ mental health and TWB.	Hwang et al. (2017), Dewaele et al. (2018), and MacIntyre et al. (2019)
Pro-social classroom behavior can increases TWB and students’ engagement, motivation, thus winning students’ appreciation and teachers’ social enjoyment.	Klassen et al. (2012), Zumbrunn et al. (2014), and Karen et al. (2018)
Students’ classroom participation and emotional input will affect teachers’ personal and social enjoyment.	Frenzel (2014), Mercer (2019), and Botes et al. (2021)
Teacher support positively correlated students’ academic emotions and achievements, which strongly predicted teaching practice, TWB and FLTE.	Butler (2012), Wang et al., 2016, Ma et al. (2017), and Lei et al. (2018)

A review of the literature on TR and TWB is instructive for second-language classroom practitioners and researchers. There are several implications on the influence of teacher education. First of all, the strong correlation between classroom emotion and motivation as well as the key role of emotional factors in the development of TR and the improvement of TWB found in the literature review suggest that more attention should be paid to the role of teachers in regulating students’ emotions, especially the internal integration of positive and negative emotions. This not only allows learners to replace negative and narrow emotions with positive and broadened emotions, but also leads to better learning outcomes (e.g., Gregersen, 2013). Meanwhile, strengthening emotional integration research also allows us to investigate more issues that fall under the category of positive psychology that have not been thoroughly studied so far, such as hope, gratitude, happiness, resilience, and character strengths (Alrabai, 2022), which are all factors associated with FLTE. In addition, integrating positive and negative emotions requires focusing on boosting FL learners’ positive emotions while working to reduce negative emotional experiences. Therefore, FL teachers should be emphasized and actively supported to effectively discover their own positive emotions in order to optimize their health, subjective wellbeing and mental resilience (Rahimi and Bigdeli, 2014), and consciously accumulate and reserve personal resources for such positive emotions, so that they can successfully extract such healthy personal emotional resource to effectively cope with challenges when coping skills are needed in difficult situations.

Second, a positive CE is likely to foster students’ classroom emotions, which can be attributed at least in part to their perception of the classroom ecology (Khajavy et al., 2017). Therefore, teachers should be encouraged to actively create a friendly and open classroom atmosphere with patient and humorous attitude by incorporating positive emotions such as care, support, encouragement, consideration, respect and enjoyment. In terms of the organization of classroom activities, teachers should strive to design and organize classroom activities with positive value and matching with students’ FL ability, and strive to provide students with a pleasant teaching context. In terms of teaching content, teachers should choose interesting teaching content that meets students’ needs to enhance the interest of FL learning, so as to improve students’ classroom participation and sense of gain. High classroom emotional atmosphere not only plays an important role in students’ emotional experience, wellbeing and academic performance, but also improves both students’ and teachers’ FLE level.

Third, FL teachers should be aware of the benefits of prosocial dynamics in the classroom. Positive interaction between teachers and students will make their relationship more harmonious, and have a positive impact on students’ academic achievement (Li, 2017) and psychological adjustment (Cornelius-White, 2007). This supportive CE increases students’ sense of belonging, engagement and motivation (Zumbrunn et al., 2014). Meanwhile, prosocial classroom behaviors will also protect teachers’ own mental health (Klassen et al., 2012). Therefore, in terms of the teaching practice supported by teachers, teachers should respect and care for students, give positive feedback to students’ classroom performance, make students feel the emotional and cognitive support of teachers, gain happiness in the FL classroom, and improve the FL learning effect. At the same time, teachers should focus on developing interpersonal skills and treat students with warmth, trust, respect and closeness, which will help teachers gain social enjoyment from students’ appreciation.

One final pedagogical implication relates to teacher training and intervention. Teacher quality is an important part of teacher education. TR and TWB have not played a role in teacher education reforms to improve the quality of teachers, although they have been ordered by the governments of many countries (Mansfield et al., 2016). In order to improve FLTE level and FL teaching quality, relieve the stress and burnout of pre-service teachers and the high attrition rate, it is very necessary to include TR and TWB in the teacher education framework. Although TR and TWB may depend on individual choices and ways of thinking, they are all intertwined with student engagement, and this review means that administrators, teachers, and teacher educators need to make TR and TWB a visible topic in the classroom, Teachers’ emotional and psychological issues need to be inserted into the curriculum of FL teacher education programs. Not only would this help pre-service teachers respond effectively to the demands of the work environment or develop some of the preparation that supports their TR and TWB, but the idea could extend to in-service teachers. For example, an initiative called “Initiatives for Teacher Wellbeing” is a 4-week online course (Ončevska and Mercer, 2019), with course structures derived from the PERMA model; Fernandes et al. (2019) proposed a training program by adjusting the modules of the European ENTRE’E scheme; Cook et al. (2017) implemented the ACHIEVER Resilience Course (ARC). All have conducted effective psychological interventions in addressing resilience, building relationship, emotional wellbeing, stress management, effective teaching and classroom management, and have achieved remarkable results in enhancing TR and TWB. In addition to such professional development programs, such as conferences, online lectures, and teacher preparation course units can serve as additional training spaces for coping strategies.

This paper gives some suggestions for further research. Future studies may further strengthen the influence of other precursors or developmental psychological variables on FLTE, so as to expand the scope of antecedents related to FL teachers’ teaching ability and enjoyment. There is already evidence that extending the antecedent study of FLE in the teachers and learners may provide more insight into its variability. For example, Proietti Ergün and Dewaele (2021) pointed out in their study that the small effect size of TWB and TR on FLTE indicated that other factors besides the study played a role in FLTE, such as institutional stress, overwork, emotional and physical exhaustion, and low income. In another meta-analysis of FLE effects, most of the summary estimates calculated by Botes et al. (2022) show high levels of heterogeneity (*I*^2^ > 0.80), suggesting that the study’s relationship with FLE may be exacerbated or hindered by other factors. It is only through additional research into FLE that these factors can be identified. From a meta-theoretical perspective, we believe that we need to find consensus and integrate the multidimensional core elements that represent enjoyment. Examples include enjoyment and satisfaction in the emotional and cognitive dimensions, satisfaction and worry in the positive and negative dimensions, and mental and physical health in the mental and physical dimensions. In terms of individual factors, in addition to the variables of resilience and wellbeing, other variables worth considering include perseverance, personality traits, emotional intelligence, mental health, and emotional regulation. However, only a few studies examined the relationship between these variables and FLE (e.g., Dewaele et al., 2019; Wei et al., 2019; Azari Noughabi et al., 2022). Furthermore, researchers ought to consider expanding positive emotion research to include variables other than FLE, such as hope, optimism and pride, as they may hold considerable promise in FL learning research (Tetzner and Becker, 2018; Botes et al., 2022).

The review found that more research is likely to be done in the future on the link between TWB and student wellbeing. Because TWB can significantly predict FLTE, emotional dynamics may be identified as contributors to emotional contagion in the classroom. Teachers’ happiness in the classroom can not only be contagious to students, but also the TWB in the classroom is affected by students’ mood. This co-construction of the experience of wellbeing in the classroom can lead to reciprocal processes and affect the FL enjoyment by both students and teachers. In terms of research results, social science research strongly recommends multi-source methods (Ham et al., 2015) to improve the validity of research results. Therefore, in order to avoid the drawbacks of relatively single source of results and mainly relying on the subjective experience and evaluation of participating teachers, the research on TR, TWB and FLTE should be considered from the dimensions evaluated by others and more situational variables to improve the validity of the research results.

In terms of environmental factors, in addition to focusing on the relationship between individual characteristics, classroom environment variables and FLTE, future studies may also pay more attention to the influence and mediating effect of other occupational environmental factors, such as institutional pressures, organizational support, campus culture and work load. The advantage of this approach is that, on the one hand, it will enable us to ensure a multi-layered research perspective on the antecedents of FLTE when investigating person-context interaction, i.e., from the micro level of the individual to the macro level of the school system, in order to enhance the validity of the findings. On the other hand, it also enables us to avoid the fundamental attribution error in social psychology, which is made by overestimating the influence of individual characteristics on behavior and underestimating situational characteristics (Kennedy, 2010).

## Author contributions

The author confirms being the sole contributor of this work and has approved it for publication.

## Conflict of interest

The author declares that the research was conducted in the absence of any commercial or financial relationships that could be construed as a potential conflict of interest.

## Publisher’s note

All claims expressed in this article are solely those of the authors and do not necessarily represent those of their affiliated organizations, or those of the publisher, the editors and the reviewers. Any product that may be evaluated in this article, or claim that may be made by its manufacturer, is not guaranteed or endorsed by the publisher.

## Abbreviations

CE: classroom environment
FL: foreign language
FLE: foreign language enjoyment
FLES: foreign language enjoyment scale
FLTE: foreign language teaching enjoyment
S-FLES: simplified foreign language enjoyment scale
TR: teacher resilience
TWB: teacher wellbeing
